# The matching rule of Panum’s limiting case and its influencing factors

**DOI:** 10.3389/fpsyg.2022.1061396

**Published:** 2023-01-06

**Authors:** Yuyu Shi, Ziyi He, Yuzhen Deng, Huayun Li

**Affiliations:** ^1^College of Teacher Education, Zhejiang Normal University, Jinhua, China; ^2^Key Laboratory of Intelligent Education Technology and Application, Zhejiang Normal University, Jinhua, China

**Keywords:** monocular occlusion region, Panum’s limiting case, matching rule, fast alternative matching, double matching

## Abstract

**Introduction:**

Panum’s limiting case is one of the typical configurations of monocular occlusion region. The matching rule of Panum’s limiting case is the key to understanding how monocular occlusion region produces stereopsis. There are currently two main views on the matching rule of Panum’s limiting case, namely double fusion and uniqueness constraint. This paper further discusses its matching mechanism on the basis of previous studies.

**Methods:**

In this study, fold line Panum’s stimuli were used to study the matching rule of Panum’s limiting case. In Experiment 1, fixation position was adopted to present the stimulus in a short time to explore the matching rules in Panum’s limiting case. In Experiment 2, the effect of fixation position on Panum’s limiting case matching results was further investigated.

**Results:**

The results of Experiment 1 show that when stimuli are presented in a short period of time, the reported result that a single feature in one eye may be matched alternately with two features in the other eye. This matching rule is called “fast alternative matching” in this article. The results of Experiment 2 results show that the position of the fixation could affect the matching result of participants.

**Conclusion:**

In conclusion, the matching rule of Panum’s limiting case is fast alternative matching, and the matching result is related to the attention state of the participant. These results not only provide a new perspective for matching rules in Panum’s limiting case, but also show that depth perception results in stereopsis can be influenced by top-down cognitive processing. This study provides a theoretical basis for studying the formation of stereopsis in the monocular region to a certain extent. In summary, the matching rule of Panum’s limiting case is fast alternative matching. In previous studies, the perceived result of double fusion may be caused by fast alternative matching. Also, the matching results are related to the participant’s state of attention, which suggests that the depth perception results of stereopsis are influenced by top-down cognitive processing.

## Introduction

**Figure 1:**
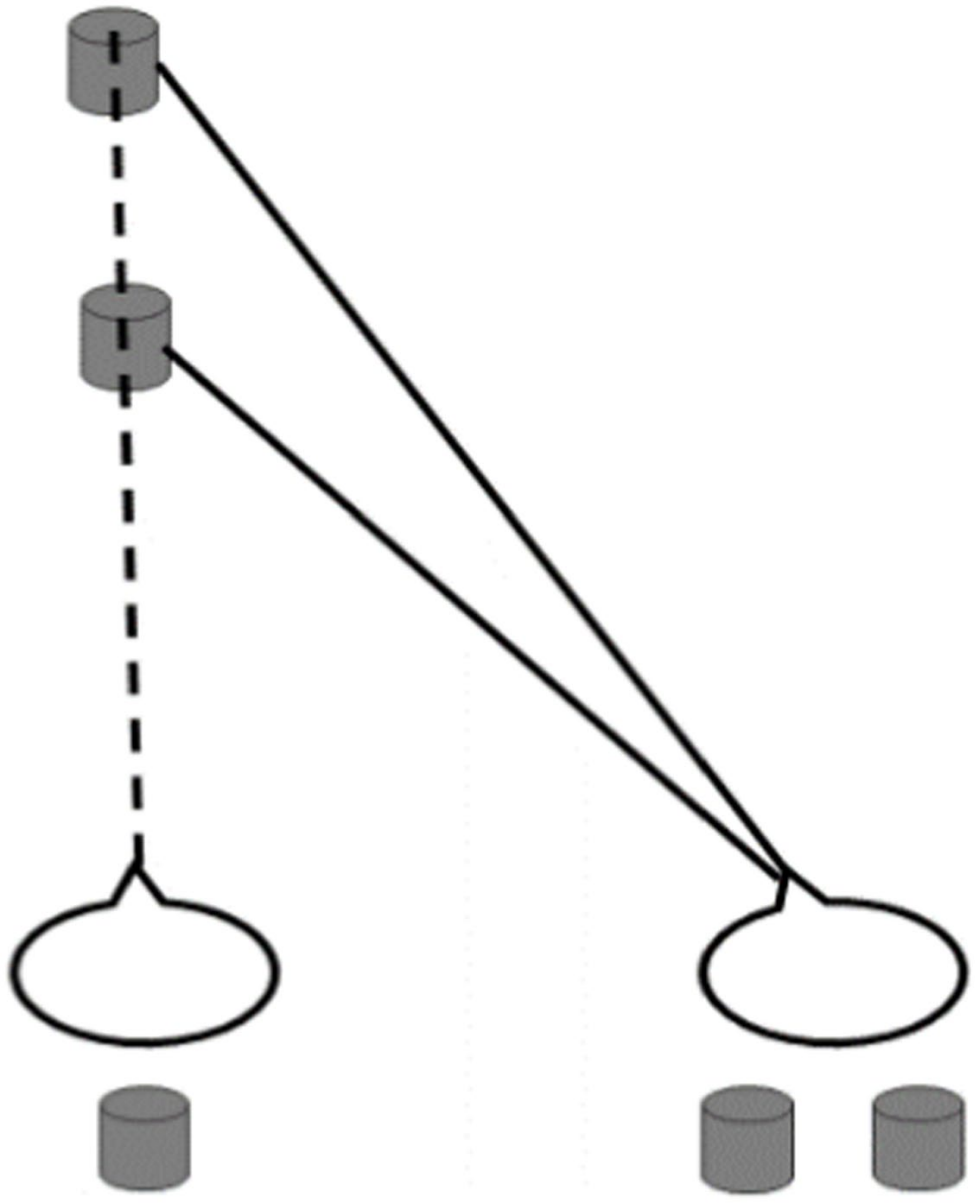
Schematic diagram for Panum’s limiting case.

As one of the earliest discovered phenomena of binocular vision in stereopsis study (Gillam and Cook, 1995), Panum’s limiting case was demonstrated by Wheatstone (1838) and Panum (1858) and characterized by one eye with one feature and the other eye with two features (as shown in Figure 1). The limiting case refers to a special case where the object in front completely occludes the object behind. In real scenes, if two opaque objects in different positions in front and behind are both at the same level as the sight line of one of the observer’s eyes, then the eye can only see one object between objects due to occlusion and the other eye can see two objects (Gettys and Harker, 1967). After binocular fusion, the observer can perceive two objects with depth (Nakayama and Shimojo, 1990; Nakamizo et al., 1994; Wang et al., 2001; Tsirlin et al., 2012; DöVencioğlu et al., 2013). For the past few decades, although Panum’s limiting case has attracted a great deal of attention, the matching rule of corresponding features in Panum’s limiting case still needs to be further illustrated (Anderson, 1994; Shimono et al., 1999; Frisby, 2001; Wang et al., 2001; Gillam et al., 2003; Cook and Gillam, 2004; Li et al., 2017).

In general, there is a certain difference in the image position of the corresponding features presented to the retina of both eyes due to the horizontal distance between the eyes, called binocular disparity (Angelis et al., 1995; Gonzalez and Perez, 1998; Alvarez et al., 2021). If the corresponding image point can be found on both retinas, this disparity in position can be retrieved, and the relative depth of the object can be measured (Tsirlin et al., 2014; Verhoef et al., 2016; Chen et al., 2021; Vishwanath, 2021). However, the presence of positional differences between the two eyes also creates monocular regions that are visible to only one eye. Therefore, the matching mechanism between the object features in the monocular regions and the object features of the other eye is controversial in Panum’s limiting case (Ono et al., 1992; Anderson and Nakayama, 1994; Shimono et al., 1999; Tsirlin et al., 2014). Some studies believe that the depth percept of Panum’s limiting case originates from double fusion (Gillam and Cook, 1995; Li et al., 2012), that is, two images on one retina and a single image on the other retina are fused together. These studies consider double fusion and double matching to be the same concept. some researchers believe that the features in one eye can match at most one of the paired features in the other eye at any time in Panum’s limiting case. This matching rule is called “uniqueness constraint” (Frisby, 2001; Howard and Rogers, 2012), which is called uniqueness matching or alternate matching in stereopsis.

There are two possible reasons for the above dispute. One is that previous studies have not taken into account that the speed at which features are matched may have an effect on the fusion rule. That is, if the speed of the alternate matching is fast enough, the participant cannot perceive the order of the alternate matching, then the perceptual result may be double fusion. For example, when an object is moving rapidly, persistence of vision phenomenon occurs (Edridge-Green, 1945; Werfel, 2011), namely, after the object disappears, the human eye retains the image of the object briefly and is able to combine the separate images. The second reason for Panum’s limiting case to be controversial is the inherent confusion between the concepts of fusion and matching. Previous researchers suggested that fusion and matching have the same neural mechanisms (Anderson and Nakayama, 1994), and that they are considered to be the same concept. Fusion emphasizes the perceptual outcome, i.e., the magnitude and direction of perceived depth (Gettys and Harker, 1967; Gillam and Cook, 1995), whereas matching emphasizes combination, i.e., features presented to one eye are combined with features presented to the other eye (Mckee et al., 1995). In a previous study, we verified the fusion pattern based on previous controversies about the fusion pattern in Panum’s limiting case, and the results supported the fusion pattern as double fusion. Therefore, in the present study, we investigated whether a single feature presented to one eye could be matched with two features presented to the other eye at the same point of time using a fixation point and a rapidly presented stimulus. Then we demonstrate whether Panum’s limiting case comes from simultaneous matching or fast alternative matching.

In addition, previous studies suggest that participants can manipulate single feature presented in one eye to match either of the two features presented in the other eye, but could not match both two features presented in the other eye in Panum’s stimulus (Gillam and Cook, 1995; Frisby, 2001). In other words, the participant can control the matching result in Panum’s stimulus by changing the position of fixation, which indicates that depth perception could be influenced by top-down processing, such as the knowledge, experience and expectation of participants (Spang et al., 2012). To our knowledge, few studies have investigated how the participant influences the matching result by controlling the position of fixation.

Based on the above consideration, the research aims to address two questions. Frist, what is the matching rule of Panum limit case? Second, does the location of fixation point affect the perception result? In the current study, we designed two experiments. In Experiment 1, we used fixation position and rapidly presented the stimulus to explore the matching rule of Panum’s limiting case. Moreover, in Experiment 2, we adopted the fixed position of fixation to investigate the effect of position of fixation on the matching result in Panum’s stimulus. We hypothesized that the matching rule of Panum’s stimulus was fast alternative matching. At the same point of time, a single feature presented to one eye can only be matched with one of the two features presented to the other eye, but not with both of features. And the position of fixation would influence the perceptual result of it. Specifically, for a single feature presented to the eye of the participant, the probability of matching with the position of fixation feature was higher than that of the position of non-fixation feature.

## Materials and methods

### Participants

There were 12 participants (7 males, aged 20–28 years, mean = 23.41, *SD* = 2.50) in Experiment 1 and eight participants (3 males, aged 20–26 years, mean = 23.75, *SD* = 1.92) in Experiment 2, all of which were local college students or graduate students. They all had normal or corrected-to-normal visual acuity above 1.0, and no other mental or organic lesions. Their stereoacuity was assessed by using the Randot™ stereotest, and the test results were required to reach 20 s of arc to participate in this study. The study was approved by the local ethics committee, and written informed consent was obtained from all participants in accordance with the Declaration of Helsinki.

### Experimental apparatus

For both Experiment 1 and 2, all visual stimuli were generated with Psychtoolbox (Brainard, 1997; Pelli, 1997) using MATLAB 7.12.0 software on Mac OS X, and observers viewed the stimuli on two DELL monitors (1920 × 1,200 pixels, 75 Hz refresh rate) in a Wheatstone stereoscope configuration. The whole experiment was carried out in a dark room with a test distance of 74 cm. To ensure that the distance between participants and the monitors remained as unchanged as possible in the experiment, participants were required to observe the stimulus by placing their chin on a chin rest and give the response by pressing keys.

### Experimental stimuli

**Figure 2:**
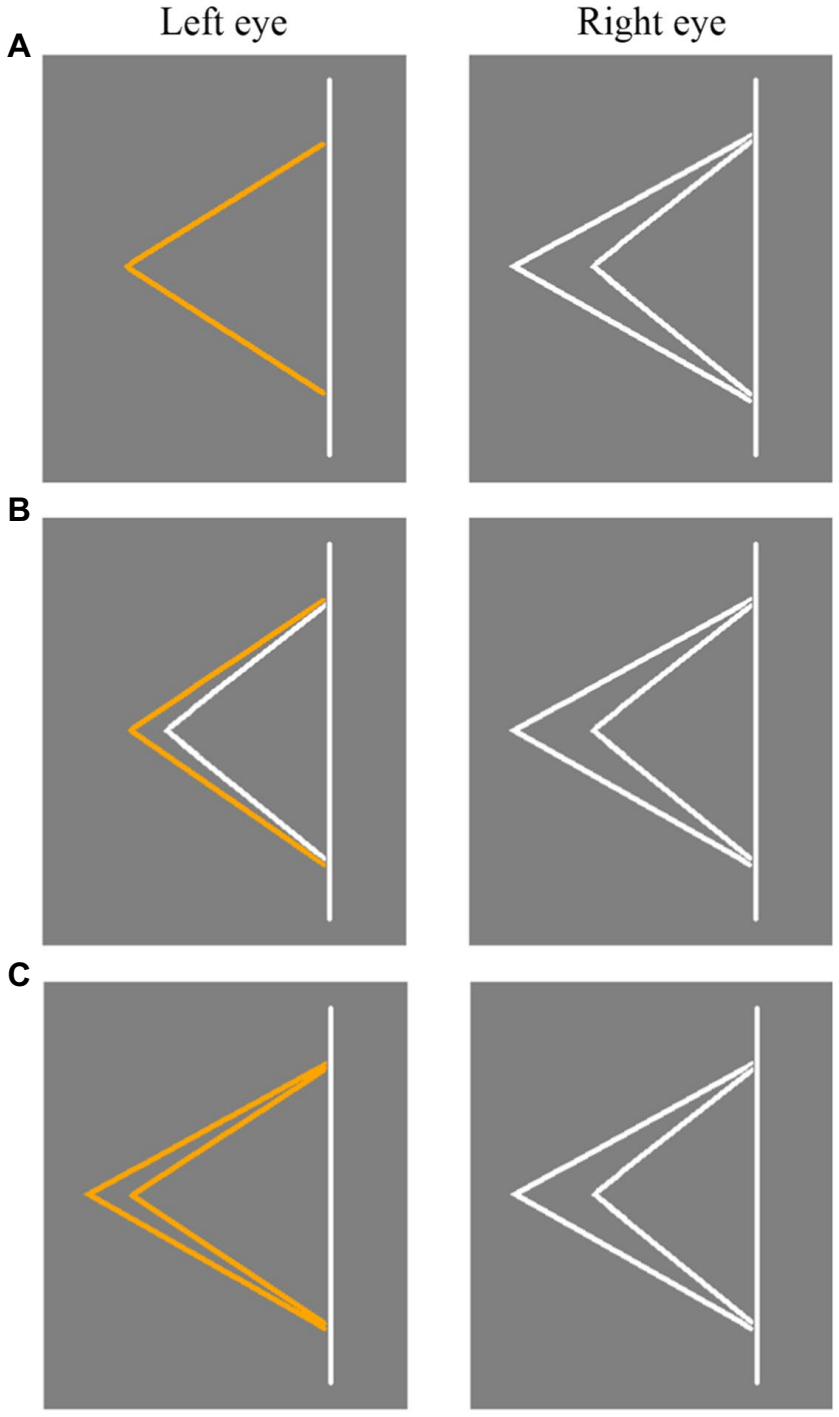
Three types of stimuli diagrams used in Experiment 1. **(A)** Panum’s stimulus. **(B)** Type I control stimulus. **(C)** Type II control stimulus. The stimulus presented to the left and right eyes was switched in different trials.

**Figure 3:**
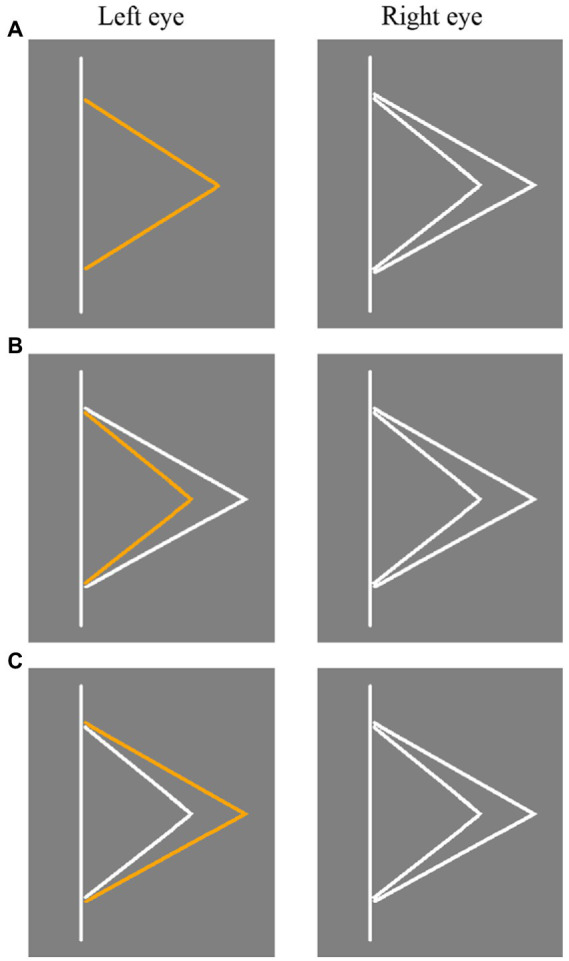
Three types of stimuli in Experiment 2. **(A)** Panum’s stimulus. **(B)** Type I control stimulus. **(C)** Type II control stimulus. The stimulus presented to the left and right eyes was switched in different trials.

The stimuli used in Experiment 1 is shown in Figure 2. Panum’s stimulus (Figure 2A) consisted of an orange fold line in one eye and two white fold lines in the other eye. Type I control stimulus (Figure 2B) included one orange and one white fold line presented to one eye and two white fold lines presented to the other eye. Type II control stimulus (Figure 2C) included two orange fold lines presented to one eye and two white fold lines presented to the other eye. In the Type I and Type II control stimuli, the range of binocular disparity was ±0.3 radians in the fixation line and 0 radian in the non-fixation line. Panum’s stimulus, Type I control stimulus, and Type II control stimulus all included four experimental conditions, i.e., the fold line was located on the left or right of the straight line. And the stimulus presented to the left and right eyes was switched in different trials. Specifically, as shown in Figures 2, 3, take Panum’s stimuli (Figure 2A) as an example, if single feature in the first column is presented to the left eye and a pair of features from the second column is presented to the right eye. Then in another trial, pairs of features in the second column are presented to the left eye and single feature in the first column are presented to the right eye, thus allowing the stimuli to be switched between the left and right eyes.

The stimulus used in Experiment 2 is shown in Figure 3. In Panum’s stimulus (as shown in Figure 3A), the stimulus presented to one eye was an orange fold line, and the distance of vertex to straight line was 1.5 radians. The stimulus presented to the other eye were two white fold lines, and the distance of vertex to the straight line was 1.2 radians and 1.8 radians, respectively. In Type I control stimulus (Figure 3B), the stimulus presented to one eye was a small orange fold line and a large white fold line, while the stimulus presented to the other eye were two white fold lines. In Type II control stimulus (Figure 3C), the stimulus presented to one eye was a small white fold line and a large orange fold line, while the stimulus presented to the other eye were two white fold lines. In Type I and Type II control stimuli, the vertex of the small fold line was 1.2 radians away from the straight line, regardless of whether the stimulus contained orange fold lines or not, and the vertex of the large fold line was 1.8 radians away from the straight line. Therefore, the binocular disparity of both the small fold line and the large fold line was 0 radian.

## Procedure

**Figure 4:**
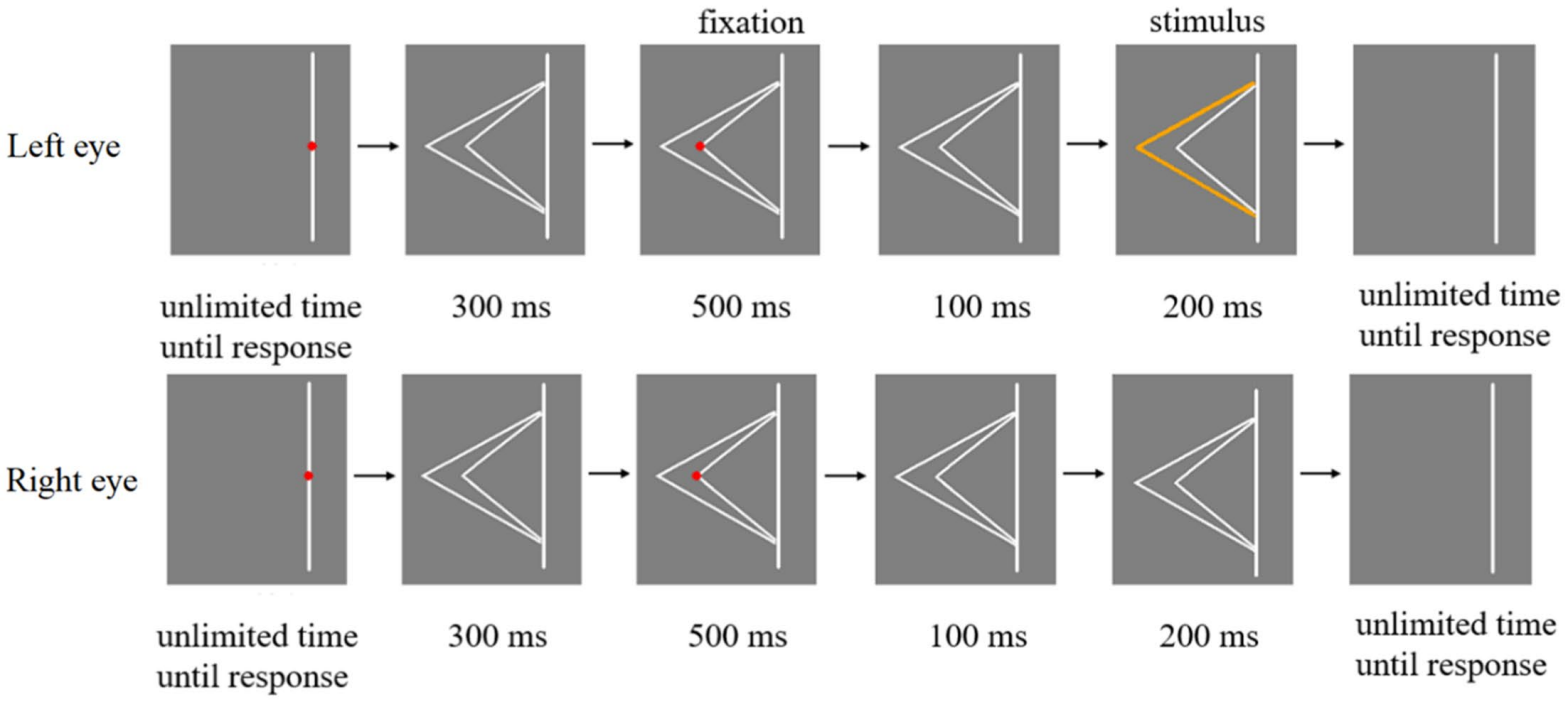
Schematic illustration of Experiment 1 procedure.

The schematic illustration of Experiment 1 procedure was shown in Figure 4. First, a red fixation point was placed in the middle of the line. The participant had to focus on the red fixation and press the space key when they were ready. Subsequently, two white fold lines were shown in eyes for 300 ms. After that, when the red fixation appeared at the top of the larger fold line (or the top of the smaller fold line), participant’s eyes should stay at the position where the fixation appeared and remain as still as possible for 500 ms. Then the red fixation disappeared and only two white fold lines were presented in eyes of the participant for 100 ms. Any one of the three types of stimuli in Figure 2 were presented in the eyes of participants for 200 ms. Finally, participants were asked to press keys to report whether they perceived one orange line or two orange lines when the stimulus was presented. Participants were asked to keep their attention at the red fixation in each trial. At the end of each trial, they were asked to report the number of orange fold line.

**Figure 5:**
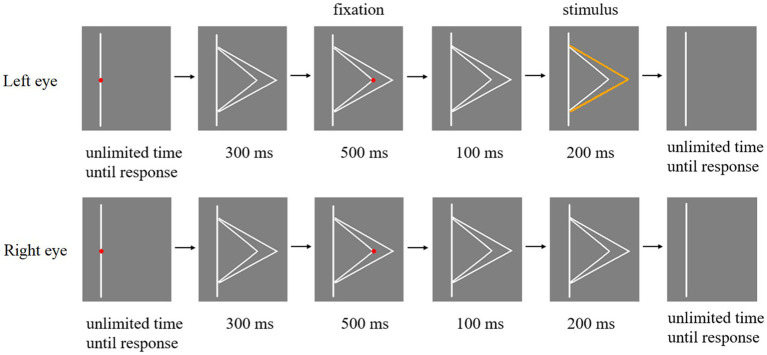
Flow chart of a trial in Experiment 2.

The experimental flow chart of Experiment 2 is shown in Figure 5. A fold line located on the right side of a line is taken as an example, and a fold line located on the left side of a line is similar. At the beginning of each trial, there was a red fixation in the middle of the straight line. Participants need to focus on this fixation and press the space key to start the experiment. Subsequently, the fixation point disappeared and two white fold lines appeared in eyes of participants for 300 ms. Then, when the red fixation appeared at the top of the large fold line (or the top of the small fold line), participants’ eyes should stay at the position where the fixation appeared and keep as still as possible for 500 ms. After that, the red fixation disappeared and only two white fold lines remained, which were displayed in eyes of the participant for 100 ms. And any one of the three types of stimuli in Figure 3 were presented to participants’ eyes for 200 ms. Finally, participants were required to report whether they perceived the orange fold line as larger or smaller fold line by pressing the button when the stimulus was presented. Then participants need to move their eyes back to the red fixation in the center of the line and ready to start the next trial.

Both Experiment 1 and Experiment 2 included a practice experiment and two blocks of formal experiment. In practice experiment, the stimuli under each experimental condition were presented twice at random. In formal experiment, for Experiment 1, the four experimental conditions of Panum’s stimulus in each block were randomly presented for 20 times, and the four conditions of control stimuli were randomly presented for 10 times within each block. For Experiment 2, eight experimental conditions of Panum’s stimulus were randomly presented for 10 times in each block, and eight experimental conditions of Type I and Type II control stimuli were randomly presented for five times. Experiment 1 and Experiment 2 each consisted of 320 trials and participants could rest between two blocks.

## Results

### Experiment 1: Speed of feature matching influence the matching rule of Panum’s limiting case

**Figure 6:**
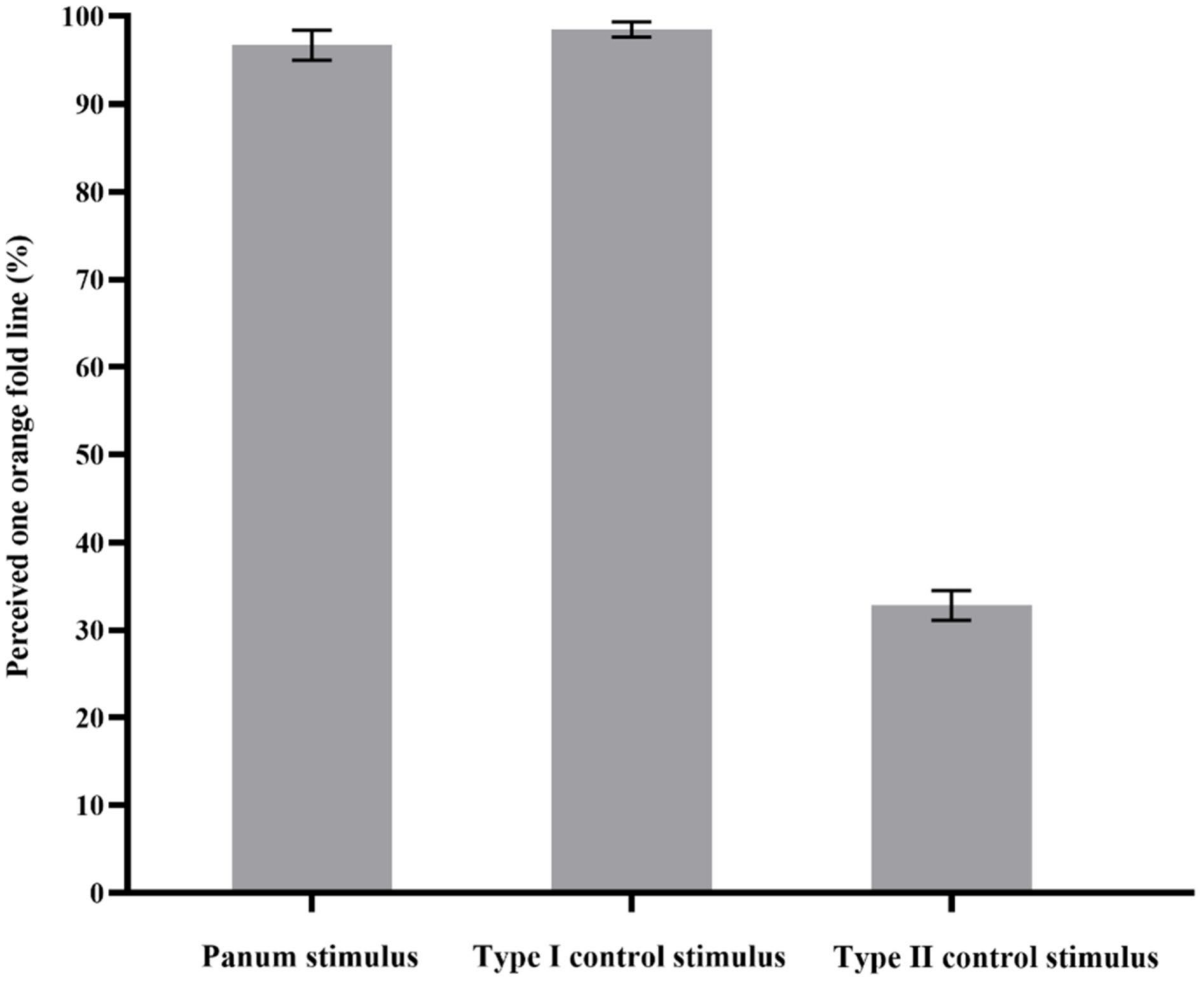
Results of Experiment 1. The y-axis indicates the probability that the participants perceived one orange fold line. The x-axis represents the three types of stimuli. The error bars in the figure refer to standard error.

The probability of participants perceived an orange fold line in Panum’s stimulus, Type I and II of control stimulus was calculated by SPSS17.0. The results showed that the main effect of the three experimental conditions was significant, *F*(1, 11) = 511.43, *p* < 0.001, η_p_^2^ = 0.98. Post-hoc tests revealed that there was no significant difference between the probability that participants perceived an orange fold line in Panum’s stimulus and Type I control stimulus (*p* > 0.05). The probability of perceiving an orange crease was significantly higher in Panum’s stimulus than in Type II control stimulus (*p* < 0.001). The probability that participants perceived an orange fold line was also significantly higher in Type I control stimulus than in Type II control stimulus (*p* < 0.001; as shown in Figure 6).

In the present study, if the probability that participant’s perceptual outcome in Panum’s stimulus was one orange fold line with no significant difference from the Type I control stimulus, then it can be inferred that the matching mechanism for Panum’s limiting case was fast alternative matching. However, if the probability that the participant’s perceptual outcome was two orange fold lines with no significant difference from the Type II control stimulus, then it can be inferred that the matching rule of Panum’s limiting case is double matching. Therefore, the above experimental results indicate that the matching rule of Panum’s limiting case is fast alternative matching.

### Experiment 2: The effect of position of fixation on matching results

**Figure 7:**
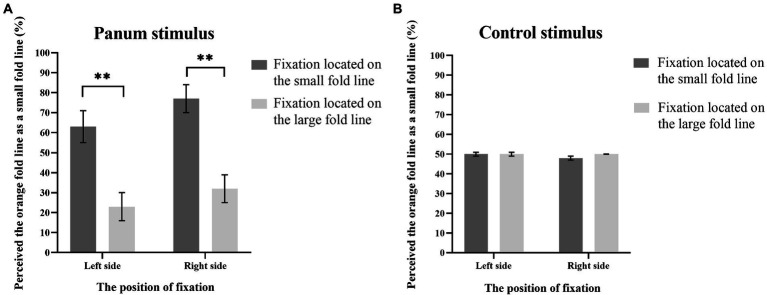
Results of Experiment 2. The y-axis indicates the probability that participants perceive the orange fold line as a small fold line under various experimental conditions, and the x-axis noted that the position of fixation. The error bars in the figures refer to standard error. The symbol “**” represents a *p*-value of < 0.005.

The results from four kinds of Panum’s stimuli and four kinds of control stimuli conditions were used for statistical analysis. The probability of participants reporting orange fold line as small fold lines under each experimental condition was calculated separately (as shown in Figure 7). 2 (position of fixation: located on the large fold line, located on the small fold line) × 2 (type of stimulus: Panum’s stimulus, control stimulus) × 2 (orientation of fold line: located on the left of the straight line, located on the right of the straight line). We used greenhouse–Geisser to correct *p* values, and Bonferroni was used to correct *p* values for pairwise comparison.

Analysis of variance found that the main effect of the position of fixation is significant, *F*(1, 7) = 20.71, *p* < 0.005, η_p_^2^ = 0.75. The main effect of orientation of fold line is significant, *F*(1, 7) = 8.34, *p* < 0.005, η_p_^2^ = 0.54. The interaction between the position of fixation and the type of stimulus was significant, *F*(1, 7) = 19.13, *p* < 0.005, η_p_^2^ = 0.73. *Post hoc* simple effect analysis showed that for Panum’s stimulus, when the fixation was on the small fold line, the probability of participants reporting the orange fold line as the small fold line was significantly higher than when the fixation was on the large fold line, *p* < 0.005. For the control stimulus, the probability that the orange dash is judged to be a small dash is not significant regardless of whether the dash is located to the left or right of the line (*p* > 0.05). The interaction between the type of stimulus and the orientation of fold line was significant, *F*(1, 7) = 13.40, *p* < 0.05, η_p_^2^ = 0.66. Simple effect analysis showed that for Panum’s stimulus, when the fold line was on the left of the line, the probability of participants reporting orange fold line as small fold line was significantly lower than when the fold line was on the right of the line, *p* < 0.05. For control stimuli, there was no significant difference in the probability of participants reporting orange fold as small fold, *p* > 0.05, regardless of whether the fold was located to the left or right of the line. Other main effects and interaction effects were not significant, *p*s > 0.05.

## General discussion

Previous studies have disputed the depth mechanism in Panum’s limiting case (Mckee et al., 1995; Gillam and Nakayama, 1999; Frisby, 2001; Wang et al., 2001; Howard and Rogers, 2012). In the present study, we revealed the matching rule of Panum’s limiting case resulted from fast alternative matching, and the position of fixation could influence the matching results. This demonstrated that the speed of feature matching had an important effect on matching rule of Panum’s limiting case. These results provide a new perspective for matching rules in Panum’s limiting case, and show that depth perception results in stereopsis can be influenced by top-down cognitive processing.

### Speed of feature matching influence the matching rule of Panum’s limiting case

The results of Experiment 1 show that although participants could perceive the depth of the two features in the Panum’s stimulus after binocular fusion, a feature presented to one eye cannot be matched simultaneously with two features presented to the other eye. These results support double fusion come from fast alternative matching. This is inconsistent with previous studies, which have supported double matching and double matching, namely double fusion (Panum, 1858; Gillam et al., 1995). The reason may be that the intervals between feature matches are too short for participants to feel that fast alternative matching is occurring. However, in this study, the presentation time of the stimulus was controlled to a short period of time. The results suggest that perceptual results of double fusion in Panum’s limiting case actually come from fast alternative matching rather than double matching.

As for the presentation time of stimulus, before the formal experiment, we referred to previous studies on stereopsis (Spang et al., 2012), and preliminarily tested how long it was appropriate to present the stimulus. Finally, we used 200 ms as the presentation time in subsequent experiments. It should be noted that if the presentation time of the stimulus is limited and the position of fixation is fixed during the presentation of the stimulus, the speed of fast alternative matching between features in different eyes would be slowed down. In this case, participants could perceive the order of fast alternative matching and be able to correctly report the perceptual results (Frisby, 2001; Wang et al., 2001). However, if the presentation time of the stimulus is unlimited and the position of fixation is not fixed, the speed of fast alternative matching between features in different eyes would be too fast for the participant to be aware of the order of the fast alternative matching. In this case, participants perceive that the matching occurs at the same point of time, and the result of double matching is perceptually supported (Gillam and Nakayama, 1999). Therefore, the present study used the same stimulus presentation time (200 ms) and controlled for the location of the fixation point in each trial, examining whether a limited stimulus presentation time and a fixation point contributed to participants’ more accurate perception of the alternate matching outcome.

### The effect of the position of fixation on matching result

The results demonstrated that participants were able to find the corresponding objects of the features presented in both eyes for control stimuli, and were able to correctly report perceptual results. It indicated that the participants did not judge the matching of features based on the position of fixation. For example, for the Type I control stimulus in Figure 3, if participants correctly matching the features presented in both eyes, they would report the orange fold line was small fold line, instead of large fold line. However, in the experimental results of Type I control stimulus, the probability that participants reported perceiving orange fold to be small fold was close to the probability expected (i.e., 50%). It indicated that whether the fixation is located on the large line or the small line, participants could not be affected by the position of fixation. And participants could report the perceptual results correctly after binocular fusion.

As for Panum’s stimulus, when the fixation was located on the small fold line, participants precepted the probability of matching a single orange feature presented in one eye with a small fold line in the other eye was significantly higher than the fixation located on the large fold line. This result shows that the position of fixation in Panum’s stimulus could affect the matching result. Specifically, compared with the feature not located at fixation, the probability of the single feature matching with the feature located at fixation in the other eye was higher. For the feature not located at fixation, subjects paid more attention to the position of fixation, suggesting that depth perception is largely dependent on the attentional state of the subjects, which is consistent with previous studies Furthermore, it suggests that depth perception could be influenced not only by the stimulus presented to the two retinas, but also by top-down processing which included the expectation of participants (Gao et al., 2021), perceptual organization (Li et al., 2017), and familiarity (Crone, 1992). The present experimental results supported this perspective, and further proved that the matching result was influenced by the top-down cognitive processing.

Based on the depth perception results in Panum’s stimulus for double fusion, this study further investigated two issues related to the matching rule of double fusion in Panum’s limiting case using fold line Panum’s stimuli. That is, whether the matching mode of double fusion can be matched at the same point of time and the extent to which the position of fixation affects the matching results. The results show that the matching rule of double fusion is fast alternative matching rather than simultaneous matching, and that the position of fixation affects the feature matching results. In summary, based on previous studies and the experimental results of this study, we propose a new theory that participants can perceive double fusion during deep processing in Panum’s limiting case. This double fusion does not come from simultaneous matching, but from fast alternative matching. In previous studies, double fusion refers to double matching or simultaneous matching, and uniqueness fusion refers to uniqueness matching or alternative matching, which are two completely opposing theories. The present study correlates double fusion with fast alternative matching for the first time, which makes up for the limitations of each of the two theories in previous studies. It resolves the long-standing and widespread controversy in Panum’s limiting case to a certain extent, and provides new evidence for establishing a better matching rule.

## Limitation and future research directions

In this study, we examined the matching rule of fusion in Panum’s limiting case. In the process of stimulus presentation, previous study pointed that if the stimulus was presented for a short time, the position of fixation could be fixed in some extent (Wang et al., 2001). However, it was still difficult to guarantee that the position of fixation could be completely fixed when the fixation disappeared. Therefore, we adopted fixation position and presented the stimulus within a transient time (200 ms) to control the position of fixation. There were still some possibilities that the position of fixation could unpredictably be changed. In future studies, eye tracker can be used to monitor the eye movement indicators of participants in real time during the experiment, which can help researchers better control the eye movement characteristics during the feature matching process. For example, in future research, if the eye movement is too large after the fixation disappeared, relevant results of the trial can be eliminated in the subsequent data analysis.

## Conclusion

In this study, Panum’s stimulus was used to investigate the effect of speed of feature matching. The results show that the matching rule of Panum’s limiting case is fast alternative matching rather than double matching. That means, a single feature presented to one eye can only be matched with one of the two features presented to the other eye, but not matched with the both features simultaneously. And attention affects perception results, because it is manipulated through the change of the position of fixation. To some extent, it provided a new perspective of the matching rule of Panum’s limiting case.

## Data availability statement

The raw data supporting the conclusions of this article will be made available by the corresponding author, without undue reservation.

## Ethics statement

The studies involving human participants were reviewed and approved by Research Ethics Committee of Zhejiang Normal University. The patients/participants provided their written informed consent to participate in this study.

## Author contributions

All authors participated in the writing of the manuscript and in the discussion of the results. HL completed the experimental design and acquired the data. YS analyzed the data and completed the manuscript. ZH and YD made contributions to revising the paper. HL provided the full guidance from the design of the experiment to the completion of the paper.

## Funding

This research was supported by the Open Research Fund of the Psychology Department of Zhejiang Normal University in 2022 and Open Research Fund of College of Teacher Education, Zhejiang Normal University (No. jykf20003).

## Conflict of interest

The authors declare that the research was conducted in the absence of any commercial or financial relationships that could be construed as a potential conflict of interest.

## Publisher’s note

All claims expressed in this article are solely those of the authors and do not necessarily represent those of their affiliated organizations, or those of the publisher, the editors and the reviewers. Any product that may be evaluated in this article, or claim that may be made by its manufacturer, is not guaranteed or endorsed by the publisher.
